# ddRADseq-assisted construction of a high-density SNP genetic map and QTL fine mapping for growth-related traits in the spotted scat (*Scatophagus argus*)

**DOI:** 10.1186/s12864-020-6658-1

**Published:** 2020-04-03

**Authors:** Wei Yang, Yaorong Wang, Dongneng Jiang, Changxu Tian, Chunhua Zhu, Guangli Li, Huapu Chen

**Affiliations:** 1 Southern Marine Science and Engineering Guangdong Laboratory (Zhanjiang), Guangdong Research Center on Reproductive Control and Breeding Technology of Indigenous Valuable Fish Species, Fisheries College, Guangdong Ocean University, Zhanjiang, 524088 China; 2 Food and Environmental Engineering Department, Yangjiang Polytechnic, Yangjiang, 529566 China

**Keywords:** *Scatophagus argus*, Linkage mapping, Quantitative trait locus, Comparative genomics, Growth-related genes, RADseq

## Abstract

**Background:**

*Scatophagus argus* is a popular farmed fish in several countries of Southeast Asia, including China. Although *S. argus* has a highly promising economic value, a significant lag of breeding research severely obstructs the sustainable development of aquaculture industry. As one of the most important economic traits, growth traits are controlled by multiple gene loci called quantitative trait loci (QTLs). It is urgently needed to launch a marker assisted selection (MAS) breeding program to improve growth and other pivotal traits. Thus a high-density genetic linkage map is necessary for the fine mapping of QTLs associated with target traits.

**Results:**

Using restriction site-associated DNA sequencing, 6196 single nucleotide polymorphism (SNP) markers were developed from a full-sib mapping population for genetic map construction. A total of 6193 SNPs were grouped into 24 linkage groups (LGs), and the total length reached 2191.65 cM with an average marker interval of 0.35 cM. Comparative genome mapping revealed 23 one-to-one and 1 one-to-two syntenic relationships between *S. argus* LGs and *Larimichthys crocea* chromosomes. Based on the high-quality linkage map, a total of 44 QTLs associated with growth-related traits were identified on 11 LGs. Of which, 19 significant QTLs for body weight were detected on 9 LGs, explaining 8.8–19.6% of phenotypic variances. Within genomic regions flanking the SNP markers in QTL intervals, we predicted 15 candidate genes showing potential relationships with growth, such as *Hbp1*, *Vgll4* and *Pim3*, which merit further functional exploration.

**Conclusions:**

The first SNP genetic map with a fine resolution of 0.35 cM for *S. argus* has been developed, which shows a high level of syntenic relationship with *L. crocea* genomes. This map can provide valuable information for future genetic, genomic and evolutionary studies. The QTLs and SNP markers significantly associated with growth-related traits will act as useful tools in gene mapping, map-based cloning and MAS breeding to speed up the genetic improvement in important traits of *S. argus*. The interesting candidate genes are promising for further investigations and have the potential to provide deeper insights into growth regulation in the future.

## Introduction

High-quality fish breed (strain) is the primary prerequisite for large-scale commercial culture. Successful aquaculture largely depends on genetic breeding for rapider growth rate, larger size, higher survival rate, better eating quality, and so on [1]. In many cultured economic aquatic animals, substantial improvement has been achieved using conventional selective breeding approaches. However, economically important traits such as growth, disease resistance, temperature tolerance and flesh quality are mostly governed by quantitative trait loci (QTLs), which are defined as chromosomal regions involving single genes or gene clusters [2]. For genetic improvement of quantitative traits, conventional breeding strategies such as family and individual selection mainly rely on the phenotype and pedigree information [3], whereas the underlying genes showing minor effects usually bring in unwanted nondeterminacy. With the big advance and increasing application of modern biotechnology, marker-assisted selection (MAS) and genomic selection using markers linked to QTLs are more effective in accelerating the genetic breeding process by improving the accuracy of selection and by speeding up genetic improvement through direct and early selection [3, 4]. As a fertile area of research on genetic breeding, QTL mapping based on genotypic data has become an important technique to facilitate the investigations on quantitative traits, and can lay an effective way to understand potential location information and numbers of linked markers for beneficial target traits [5].

A genetic linkage map is a helpful tool possessing tremendous potential to facilitate QTL mapping for target traits with economic values as well as genomics and genetics studies, including map-based cloning, comparative genome analysis, and whole-genome assembly [3]. For aquaculture fishes, two genetic linkage maps were first constructed in *Oncorhynchus mykiss* [6] and *Oreochromis niloticus* [7] around the same time. And during the past 20 years, genetic breeding experts have constructed numbers of genetic maps utilizing various types of molecular markers, such as AFLP (amplified fragment length polymorphism), RAPD (random amplified polymorphic DNA) and SSR (simple sequence repeat), in many kinds of aquaculture animals, including over 30 fish species. However, a majority of existing linkage maps have low marker density, their abilities to assist in the fine-scale mapping of QTLs and other studies were seriously limited. Compared with other types of marker, single nucleotide polymorphisms (SNPs) can be genotyped on a more abundant and much larger scale [8], which has become the most popular type of codominant marker for the construction of genetic maps with higher marker-density and resolution. Due to the high cost and laborious work for SNP genotyping, however, there was a big challenge to obtain a large number of SNPs and genotype in relative large mapping families [9]. Benefiting from the rapid development of next-generation sequencing (NGS) technology in the past decade, varieties of genotyping-by-sequencing (GBS) techniques have been created and widely employed in time-saving and cost-effective SNP markers discovery and genotyping throughout the genome, even in non-model species [10]. Of these methods, restriction site-associated DNA sequencing (RADseq) and its derivative methods ddRAD [11], SLAF [12] and 2b-RAD [13] have been successfully utilized for high density (HD) linkage maps construction in many fish species, such as *Scophthalmus maximus* [14], *Salmo salar* [15], *Paralichthys olivaceus* [16], *O. niloticus* [17], and *Lates calcarifer* [18].

As one of the most important quantitative traits controlled by multi-gene QTLs as well as environmental factors, fish growth can directly affect the yield of aquaculture [19]. Delightedly, QTL mapping enables us not only to detect genetic markers associated with the genetic variation for important traits but also to find out the candidate genes involving in the regulatory processes of target traits [9]. Up to now growth-related traits have been mapped and well-studied in a wide variety of fish species with economic importance. Significant QTLs associated with growth traits have been identified, and in most cases growth-related QTLs are distributed on multiple linkage groups, e.g., 14 QTLs on 8 LGs in Yellow River carp [1], 21 QTLs on 12 LGs in Yangtze River common carp [3], 28 QTLs on 5 LGs in *Pseudobagrus ussuriensis* [9], 6 QTLs on 6 LGs in *L. calcarifer* [18], and 23 QTLs on 4 LGs in *Trachinotus blochii* [20]. These research findings have been greatly accelerating the progress of genetic improvement in economic fishes via providing powerful tools for MAS breeding.

The spotted scat *Scatophagus argus* (order Perciformes, family Scatophagidae) generally inhabits around the Indo-Pacific region, including southeast China [21]. Owing to its notable features such as high nutritional value, easy cultivation, low feeding cost and strong disease resistance, *S. argus* has become a popular aquaculture fish species in southeast Asia [22]. According to an incomplete survey, it has become a valuable species presently and been widely cultured in Guangdong, Guangxi, and Taiwan provinces of China with an annual output value of approximately RMB 150 Million. The commercial demand for seedlings has constantly grown over recent years. In view of its economic importance, *S. argus* has been intensively studied on reproductive biology, especially on artificial inducing [23–27] and mechanism of reproductive regulation [28–33] in recent years. Artificial propagation studies have been carried out since the year 2003 in China [34] and fortunately, a highly efficient technique had been established several years ago [25, 35]. However, few genetic and genomic studies have been reported for *S. argus* yet. As with many other farmed fish, the serious lag of breeding research and declining population resource have resulted in certain regressions in growth traits and disease resistance of cultured fish, which can seriously impact the quality and safety of food fish products. Hence it is urgent to launch a breeding program to promote the sustainable development of *S. argus* fish industry by improving important genetic traits.

In this study, we applied double digest restriction site-associated DNA sequencing (ddRADseq) method to identify thousands of high-quality polymorphic SNP markers by genotyping a full-sib mapping family of *S. argus*. Then linkage mapping and QTL analysis were performed. The main purposes of our study were to obtain a HD SNP-based genetic map, identify a number of growth-related QTLs with large effects and significant markers for possible use in MAS, and to provide potential genes for further studies on regulatory mechanism of growth.

## Results

### Phenotypic analysis of growth-related traits

Eight growth-related traits of the mapping family consisting of 420 full-sib progeny were measured and investigated. Kolmogorov-Smirnov tests were performed and the results indicated that these measured traits were totally in concordance with normal distribution (*P* > 0.05). The phenotypic variations and frequency distribution of these growth traits are shown in Additional file 1: Table S1 and Additional file 2: Figure S1. The mean values ± SD of BW, TL, BL, BH, HL, PD, PA and CPH were 81.906 ± 17.751 g, 14.605 ± 0.955 cm, 12.399 ± 0.849 cm, 7.100 ± 0.552 cm, 3.051 ± 0.251 cm, 3.723 ± 0.342 cm, 8.183 ± 0.600 cm and 1.585 ± 0.120 cm, respectively. Their phenotypic values displayed abundant variations, especially for BW, in which the highest coefficient of variation (21.67%) was observed. Pearson’s correlation analysis was also conducted and all growth-related traits showed a significant correlation with each other (*r* = 0.540 ~ 0.993, *P* < 0.001) (Table 1). Specifically, BW significantly correlated with BH (*r* = 0.872), BL (*r* = 0.865) and TL (*r* = 0.864). The highest correlation coefficient value was observed between BL and TL (*r* = 0.993), followed by that between BH and BL (*r* = 0.906), while the weakest correlation occurred between PD and BH (*r* = 0.540).

**Table 1:** Pearson’s correlation coefficients for all pairwise combinations of the eight growth-related traits of spotted scat F1 full-sib family (*P* < 0.001 for all)

Traits	BW	TL	BL	BH	PD	PA	HL	CPH
BW	1	0.864	0.865	0.872	0.627	0.789	0.634	0.817
TL		1	0.993	0.902	0.625	0.831	0.644	0.836
BL			1	0.906	0.624	0.841	0.662	0.832
BH				1	0.540	0.786	0.614	0.811
PD					1	0.703	0.708	0.630
PA						1	0.742	0.859
HL							1	0.661
CPH								1

*BW* body weight, *TL* total length, *BL* body length, *BH* body height, *PD* pre-dorsal length, *PA* pre-anal length, *HL* head length, *CPH* caudal peduncle height.

### ddRAD libraries sequencing

The ddRAD libraries of two parents and their 200 full-sib offspring were sequenced on an Illumina Hiseq2500™ platform. A total of 1753,714,542 raw reads (150 bp in length) were obtained, comprising approximately 257.8 Gb sequencing data (Table 2). The average reads number of the standard libraries for two parents was 59,071,871, whereas that of each progeny was 8,177,854. The sequencing depth of each parent and progeny reached an average of 14.5× and 2.0×, respectively. Subsequent trimming, quality filtering and low-quality reads removing finally generated 1,737,252,420 clean reads in total. The female and male parental data contained 56,800,034 filtered reads with a Q20_Rate of 96.77% and 60,536,986 filtered reads with a Q20_Rate of 96.74%, respectively. An average of 8,099,577 clean reads was produced for each individual of the offspring, which was equivalent to approximate 1.19 Gb of data.

**Table 2:** Statistic summary of the ddRADseq data for the mapping population of spotted scat

Type	Item	Female parent	Male parent	Average of progeny
Raw data	GC_Rate (%)	40.17	40.16	40.66
	Q20_Rate (%)	96.77	96.74	94.71
	Q30_Rate (%)	91.87	91.85	89.35
	Raw reads	57,224,366	60,919,376	8,177,854
	Raw base (bp)	8,411,981,802	8,955,148,272	1,202,173,359
	Depth	195.10×	207.69×	27.88×
Clean data	GC_Rate (%)	40.12	40.12	40.58
	Q20_Rate (%)	96.77	96.74	94.75
	Q30_Rate (%)	91.87	91.84	89.40
	Clean reads	56,800,034	60,536,986	8,099,577
	Clean base (bp)	8,345,222,442	8,894,041,121	1,189,864,968
	Effective data rate (%)	99.21	99.32	98.95

### SNP detection and genotyping

Based on ddRADseq of the *S. argus* mapping family and bioinformatics analysis, a total of 88,789 original polymorphic markers were detected using the STACKS pipeline. Through stringent screening, 20,921 high-quality polymorphic SNP markers were successfully genotyped in both parents and at least 90% of the offspring (Additional file 3: Table S2). Of which, 17,663 (84.43%) parent-specific SNP loci were heterozygous in either of the parents, and 3258 (15.57) SNPs were heterozygous in both of the parents. After segregation distortion tests, 6196 (29.62%) SNPs that were consistent with a Mendelian segregation pattern (*P* ≥ 0.01) were finally retained and utilized in the following linkage analysis (Table 3). All Mendelian SNPs were classified into three categories based on their segregation types, the marker numbers for maternal heterozygosity (lm × ll) and paternal heterozygosity (nn × np) were 2566 (41.41%) and 2683 (43.30%), respectively; and the remaining 947 (15.28%) markers were heterozygous in both parents (hk × hk and ef × eg).

**Table 3:** Statistic information of Mendelian SNP markers showing heterozygosity in one or both parents

Segregation patterns	Segregation ratio	Number of SNP loci	Ratio (%)
hk × hk	1:2:1	932	15.04
lm × ll	1:1	2566	41.41
nn × np	1:1	2683	43.30
ef × eg	1:1:1:1	15	0.24
ab×cd	1:1:1:1	0	0
Total	\	6196	\

### Construction of genetic linkage maps

**Fig. 1:**
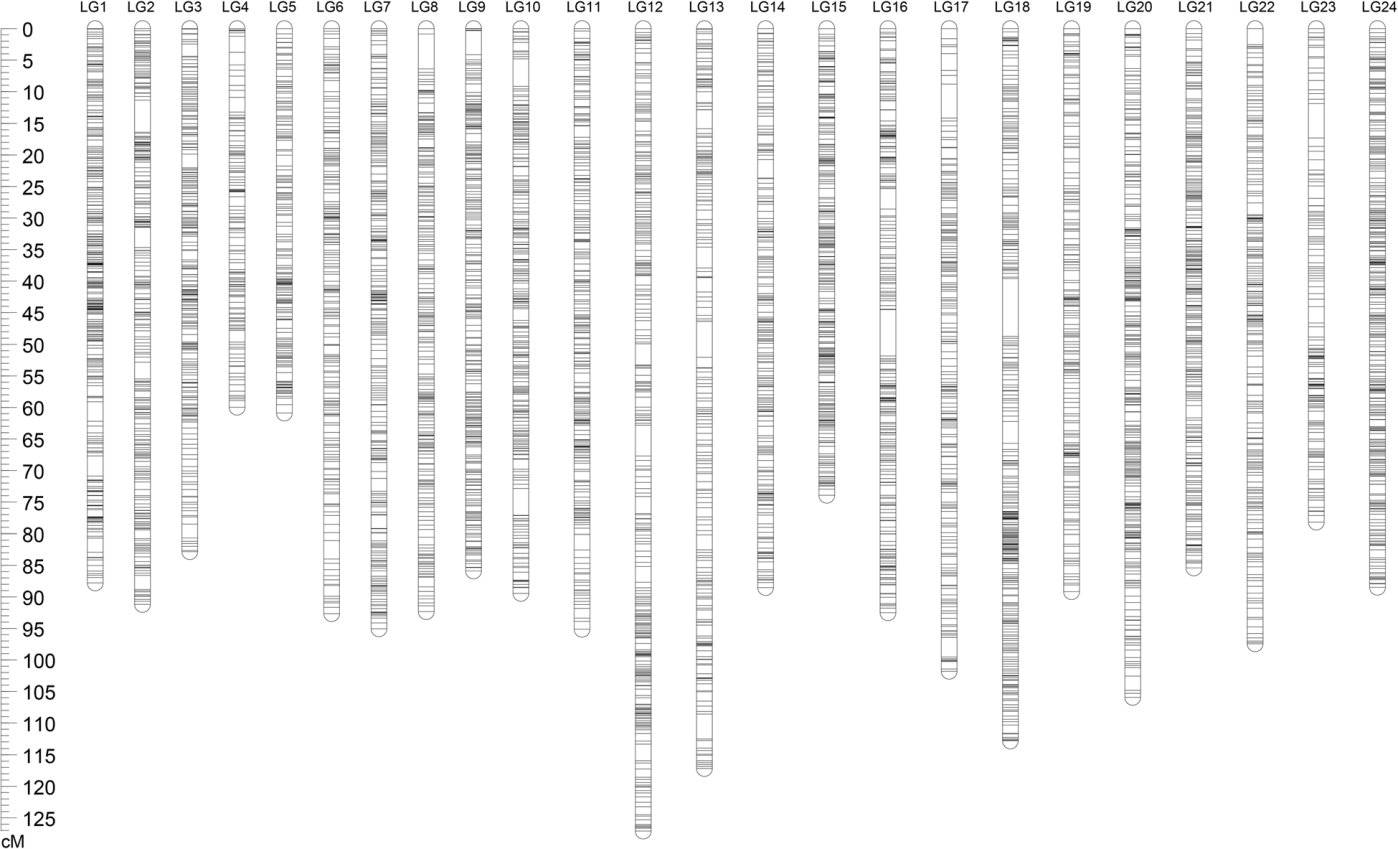
Illustration of the high-density SNP consensus linkage map of *S.argus*. The map demonstrates the genetic lengths and marker distribution of 6193 SNP loci along the 24 linkage groups (LG1 - LG24). The linkage groups are displayed by the vertical bars with black lines in each linkage group indicating a marker position. Genetic distance is shown by the vertical scale line with centiMorgans (cM)

Using the JoinMap 4.1 software with a LOD threshold of 8.0, a consensus genetic map was constructed. A total of 6193 (99.9%) out of the 6196 polymorphic SNP markers were successfully grouped into 24 linkage groups (LGs), spanning a total length of 2191.65 cM with an average marker interval of 0.35 cM (Table 4 and Fig. 1). The number of LGs is perfectly consistent with the diploid chromosome number of *S. argus* (2 *N* = 48) [36]. The number of mapped markers in each LG varied from 137 (LG4) to 351 (LG20) with an average of 258 SNPs per group. The longest LG was 127.09 cM (LG12) in length and the shortest group was only 59.96 cM (LG4) in length, whereas average intervals between two adjacent markers ranged from 0.24 cM (LG15) to 0.58 cM (LG13). Based on two commonly-used estimating methods [37, 38], the expected genome length was estimated to be 2209.20 cM (G_e1_) and 2209.31 cM (G_e2_), with an average of 2209.25 cM (G_e_). Herein the genome coverage of this linkage map reached 99.2% (Additional file 7: Table S4). As the genome size of *S. argus* has been estimated to be 598.73 Mb (unpublished data), the average recombination rate across all LGs was ~ 3.7 cM per Mb.

**Table 4:** Summary of the SNP-based high-density genetic map of spotted scat

Linkage group	Number of markers	Genetic length (cM)	Marker interval (cM)	Maximum gap (cM)
LG1	316	87.78	0.28	3.20
LG2	254	91.20	0.36	5.03
LG3	252	82.83	0.33	2.18
LG4	137	59.96	0.44	2.56
LG5	193	60.89	0.32	1.74
LG6	218	92.66	0.43	2.91
LG7	285	95.06	0.33	2.12
LG8	260	92.34	0.36	5.52
LG9	295	85.88	0.29	3.81
LG10	276	89.44	0.32	4.38
LG11	278	95.13	0.34	2.44
LG12	291	127.09	0.44	4.89
LG13	202	117.17	0.58	5.71
LG14	236	88.52	0.38	2.91
LG15	305	73.94	0.24	1.46
LG16	232	92.51	0.40	7.27
LG17	231	101.80	0.44	5.33
LG18	327	112.81	0.34	9.09
LG19	216	89.17	0.41	1.76
LG20	351	105.94	0.30	2.28
LG21	310	85.41	0.28	1.95
LG22	238	97.44	0.41	2.57
LG23	150	78.19	0.52	5.38
LG24	340	88.48	0.26	1.52
Maximum	351	127.09	0.58	9.09
Minimum	137	59.96	0.24	1.46
Total	6193	2191.65	8.79	87.99
Average	258	91.32	0.35	3.67

Two sex-specific maps each consisting of 24 LGs were also constructed (Additional file 4: Table S3, Additional file 5: Figure S2 and Additional file 6: Figure S3). The female map spanned a total length of 2290.56 cM with an average inter marker distance of 0.65 cM, whereas the male map spanned a total genetic distance of 1880.23 cM with an average marker interval of 0.52 cM. The genetic length of individual LGs of female and male maps varied from 43.12 cM (LG4) to 137.55 cM (LG12) and from 44.49 cM (LG4) to 126.56 cM (LG12), respectively. In order to validate the quality of genetic maps, synteny analyses between the consensus map and female or male map were performed. The syntenic relationships of shared markers between the consensus map and sex-specific maps were highly consistent (Additional file 8: Figure S4).

### Comparative genome mapping

**Fig. 2:**
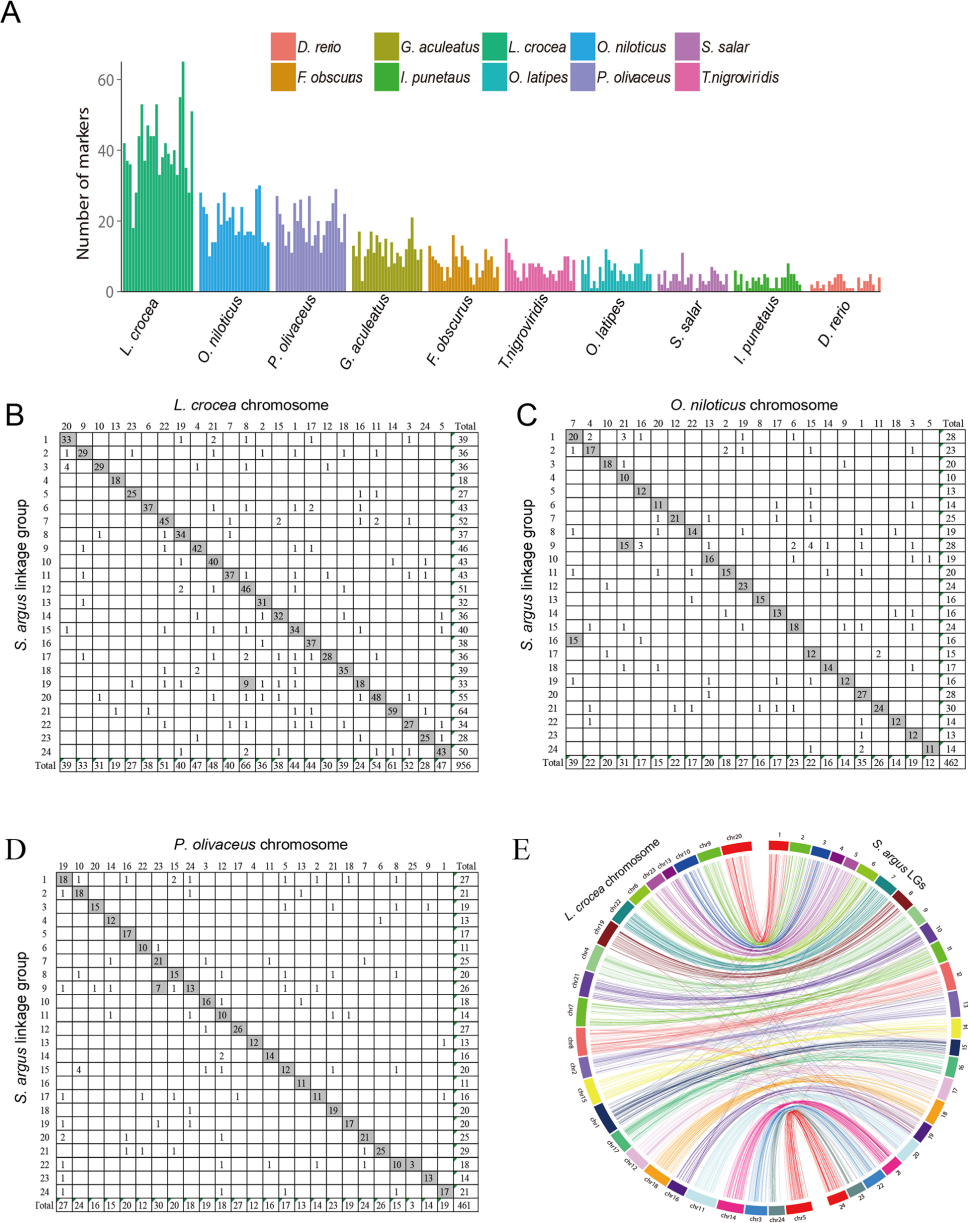
Comparative genomics analysis for the genetic map of *S.argus*. (**a**) the numbers of markers per linkage group that showed homology with genome sequences of other fish, listed on the x-axis; (**b**)-(**d**) Oxford grids showing conservation of macrosynteny relationships between *S.argus* linkage groups and chromosomes of *L. crocea* (**b**), *O. niloticus* (**c**), and *P. olivaceus* (**d**). The numbers of *S.argus* ddRAD markers with significant BLASTn hits in the other fish are presented in box, and the putative syntenic pairs are indicated by gray boxes along the diagonal. (**e**) Circos diagram representing syntenic relationships between proposed linkage groups of *S.argus* (right) and published chromosomes of *L. crocea* (left). only markers on each linkage group of *S.argus* that were mapped to a single chromosome of *L. crocea* were illustrated. Each colored arc represents a marker match between linkage group and chromosome

Successful construction of *S.argus* genetic map provided a framework to compare its conserved genomic regions with those of other teleosts. Homology searches against the genomes of 10 model or non-model fishes were explored using the ddRAD loci mapped in *S.argus* genetic map (Fig. 2). The fewest number of homologous ddRAD loci were observed in the comparison with *D. rerio* (56), followed by comparisons with *I. punetaus* (77) and *S. salar* (94). Homologous sequences to more than 400 *S. argus* ddRAD loci were found in *L. crocea* (978), *O. niloticus* (472), and *P. olivaceus* (463) (Fig. 2a). Moreover, Oxford grids were made for *S. argus* against above three teleosts based on the number of orthologous markers on each LG or chromosome. All 24 pairs of LGs or chromosomes in *S. argus* and *L. crocea* showed a basically clear 1:1 syntenic relationship (Fig. 2b), indicating a relatively high-level of genomic synteny between these two species. Comparisons with the other two fish species also indicated highly conservative 1:1 relationships, although several 1:2 syntenic relationships were observed across the genomic regions (Fig. 2c and d). For example, *O. niloticus* chromosome 7 corresponded to LG1 and LG16 in *S. argus* (Fig. 2c). *P. olivaceus* chromosome 23 corresponded to LG7 and LG9 in *S. argus* (Fig. 2d). Of these fish species analyzed, *L. crocea* exhibited by far the closest phylogenetic relationship with *S. argus*. *L. crocea* chromosomes appear to show a high degree of syntenic relationship with the *S. argus* LGs, as every chromosome is clearly linked to one linkage group in *S. argus* with an exception of chromosome 8 (Fig. 2b). Of the 956 markers uniquely anchored to *L. crocea* chromosomes, 832 (87.0%) were located into syntenic boxes (Fig. 2e). On the whole, there is a strong correlation of each *S. argus* linkage group to a single chromosome not only in *L. crocea* but also in *O. niloticus*, which are both belong to Perciformes. Our investigation primarily validated the reliability of *S. argus* linkage map, which will establish informative genome resources for future studies.

### QTL analysis for growth traits

**Fig. 3:**
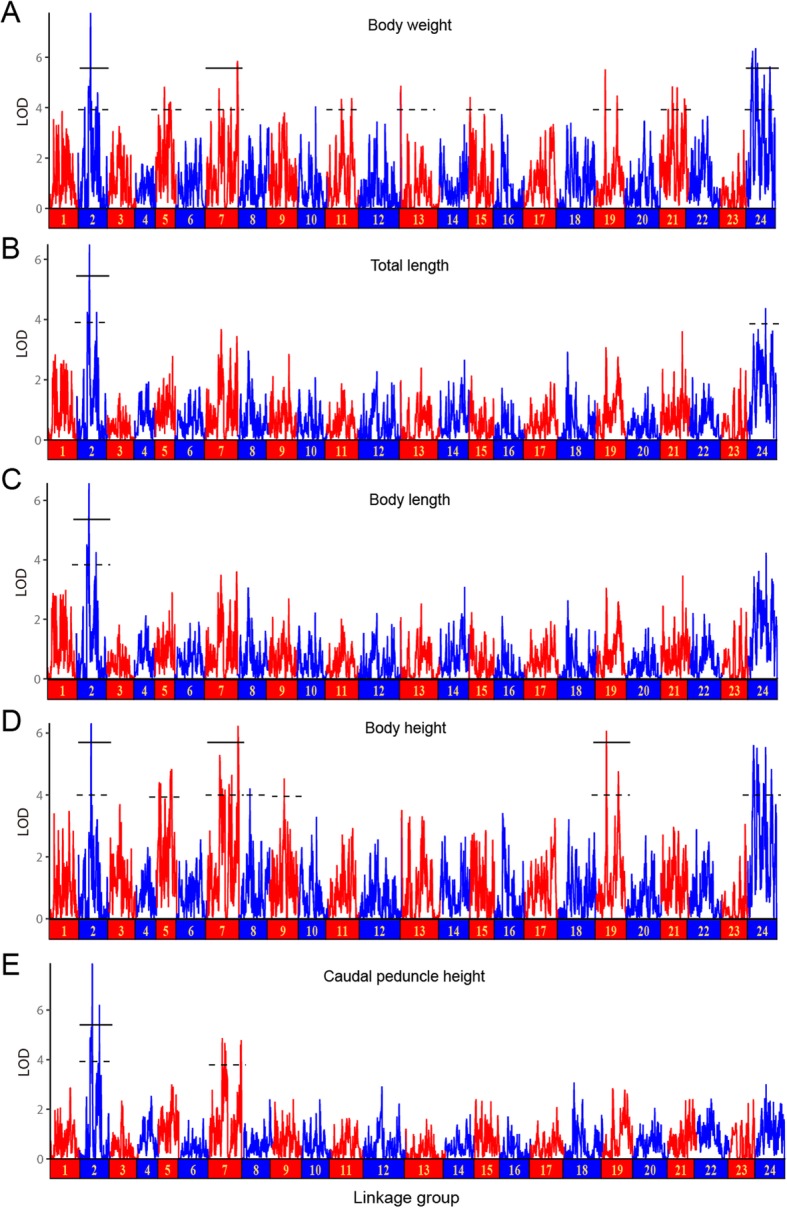
LOD curves of growth-related QTL mapping for (**a**) body weight, (**b**) total length, (**c**) body length, (**d**) body height, and (**e**) caudal peduncle height in *S.argus*. The dashed and solid lines indicated the chromosome-wide and genome-wide significance thresholds, respectively. LOD significance threshold levels were determined on the basis of 1000 permutations at a significance level of 0.05. The horizontal axes represent linkage groups LG1 - LG24

According to the Pearson’s correlation coefficients, five growth traits (BW, TL, BL, BH and CPH) showing relatively high correlation with each other were selected for QTL analysis in this study. As determined by permutation tests, the estimated values of chromosome-wide (CW) and genome-wide (GW) significance thresholds for growth-related traits varied from 3.4 to 3.8 and 5.3 to 5.4, respectively. By using MQM method in MapQTL 5.0, a total of 44 QTLs associated with growth traits were detected on 11 LGs, including 17 GW significant QTLs and 27 CW significant QTLs with LOD scores ranging from 4.21 to 7.88 (Table 5 and Fig. 3). LG24 had the highest number of QTLs (11), followed by LG2 (10) and LG7 (8), while LG8, LG9, LG13 and LG15 each only contained one. A total of 19 QTLs associated with body weight were distributed on 9 LGs (LG2, LG5, LG7, LG11, LG13, LG15, LG19, LG21 and LG24) with phenotypic variance explained (PVE) values ranging from 8.8% (qBW15–1) to 19.6% (qBW2–1). Meanwhile, 14 significant QTLs for body height were detected on 7 LGs (LG2, LG5, LG7, LG8, LG9, LG19 and LG24) with PVE values varying from 9.7% (qBH8–1) to 16.5% (qBH2–1). Five QTLs associated with CPH (qCPH2–1, qCPH2–2, qCPH7–1, qCPH7–2 and qCPH7–3) were located at 37.607 cM, 58.917 cM along LG2, and 37.527 cM, 45.350 cM, 94.126 cM along LG7, accounting for 18.5, 13.3, 11.1, 10.7 and 11.0% of the phenotypic variations, respectively. Interestingly, the QTL with relatively higher PVE values were all located at intervals on LG2, e.g., 30.983–40.473 cM for BW, 30.708–37.607 cM for CPH, and 36.998–38.312 cM for TL, BL and BH, suggesting that LG2 may play a more important role in growth regulation in *S. argus*. Specifically, the peak LOD values of QTLs associated with BW, TL, BL, BH and CPH were located at 37.607 cM of LG2 near the SNP marker R1_98424, contributing to 19.6, 16.4, 16.9, 16.5, and 18.5% of the phenotypic variation, respectively (Table 5). Because of the high correlation value (*r* = 0.993) between BL and TL, most QTLs for these two traits were located at the overlapped confidence intervals along LG2.

**Table 5:** Summary of the results of linkage mapping for QTLs associated with growth traits

Traits	QTL	LG	CI (cM)	Nearest marker	Position (cM)	LOD	PVE (%)	Significance level
BW	qBW2–1	2	30.983–40.473	R1_98424	37.607	7.76	19.6	GW
	qBW2–2	2	58.402–59.763	R1_192243	58.917	4.61	10.1	CW
	qBW5–1	5	25.992–27.809	R1_269416	25.992	4.83	11.2	CW
	qBW7–1	7	36.461–39.134	R1_85816	37.461	4.77	10.9	CW
	qBW7–2	7	91.849–95.060	R1_211226	93.370	5.85	13.4	GW
	qBW11–1	11	43.640–45.338	R1_205729	43.64	4.35	9.6	CW
	qBW11–2	11	71.138–76.128	R1_121023	74.822	4.38	9.6	CW
	qBW13–1	13	0–0.804	R1_113228	0.543	4.87	10.7	CW
	qBW15–1	15	2.747–5.174	R1_162345	3.972	4.42	8.8	CW
	qBW19–1	19	30.002–30.806	R1_140416	30.237	5.52	12.8	GW
	qBW19–2	19	63.688–67.454	R1_198192	66.225	4.48	10.2	CW
	qBW21–1	21	37.238–41.709	R1_68531	37.567	4.84	10.6	CW
	qBW21–2	21	50.546–53.101	R1_71627	52.125	4.81	11.2	CW
	qBW24–1	24	13.099–19.843	R1_255417	18.653	6.25	13.7	GW
	qBW24–2	24	26.409–34.608	R1_77061	27.503	6.36	14.6	GW
	qBW24–3	24	46.786–50.373	R1_137885	47.525	4.76	10.6	CW
	qBW24–4	24	53.208–53.942	R1_70232	53.435	5.31	11.6	GW
	qBW24–5	24	68.218–71.651	R1_175083	70.827	5.63	12.3	GW
	qBW24–6	24	73.194–74.727	R1_227285	73.194	4.49	13.4	CW
TL	qTL2–1	2	36.998–38.312	R1_98424	37.607	6.5	16.4	GW
	qTL2–2	2	58.475–59.267	R1_192243	58.917	4.25	9.3	CW
	qTL24–1	24	53.208–53.942	R1_70232	53.435	4.38	9.7	CW
BL	qBL2–1	2	30.983–35.899	R1_91299	31.482	4.46	11.7	CW
	qBL2–2	2	36.998–38.312	R1_98424	37.607	6.58	16.9	GW
	qBL2–3	2	58.402–59.267	R1_192243	58.917	4.26	9.3	CW
BH	qBH2–1	2	36.998–38.312	R1_98424	37.607	6.32	16.5	GW
	qBH5–1	5	5.054–9.952	R1_291555	8.877	4.41	9.8	CW
	qBH5–2	5	41.779–47.428	R1_129989	46.184	4.84	10.8	CW
	qBH7–1	7	37.461–43.712	R1_85816	37.461	5.29	12.1	CW
	qBH7–2	7	72.254–74.31	R1_61604	73.623	4.65	10.3	CW
	qBH7–3	7	92.377–95.06	R1_92737	92.439	6.23	13.6	GW
	qBH8–1	8	32.209–33.716	R1_49419	33.402	4.21	9.7	CW
	qBH9–1	9	42.675–44.714	R1_101372	44.120	4.53	9.9	CW
	qBH19–1	19	30.002–32.092	R1_140416	30.237	6.07	14.2	GW
	qBH19–2	19	63.418–69.448	R1_198192	66.225	4.76	10.8	CW
	qBH24–1	24	14.585–19.843	R1_129529	17.156	5.61	12.3	GW
	qBH24–2	24	26.409–34.608	R1_77061	27.503	5.52	13.1	GW
	qBH24–3	24	53.208–53.942	R1_115171	53.435	5.54	12.2	GW
	qBH24–4	24	68.218–74.727	R1_175083	70.827	4.83	11.3	CW
CPH	qCPH2–1	2	30.708–37.607	R1_98424	37.607	7.88	18.5	GW
	qCPH2–2	2	58.475–59.267	R1_192243	58.917	6.21	13.3	GW
	qCPH7–1	7	37.461–38.198	R1_85816	37.527	4.87	11.1	CW
	qCPH7–2	7	42.505–53.388	R1_235992	45.35	4.67	10.7	CW
	qCPH7–3	7	91.849–95.06	R1_93312	94.126	4.79	11	CW

*LG* Linkage group, *CI* Confidence interval, *GW* Genome-wide significance, *CW* chromosome-wide significance, *PVE* Phenotypic variance explained

**Fig. 4:**
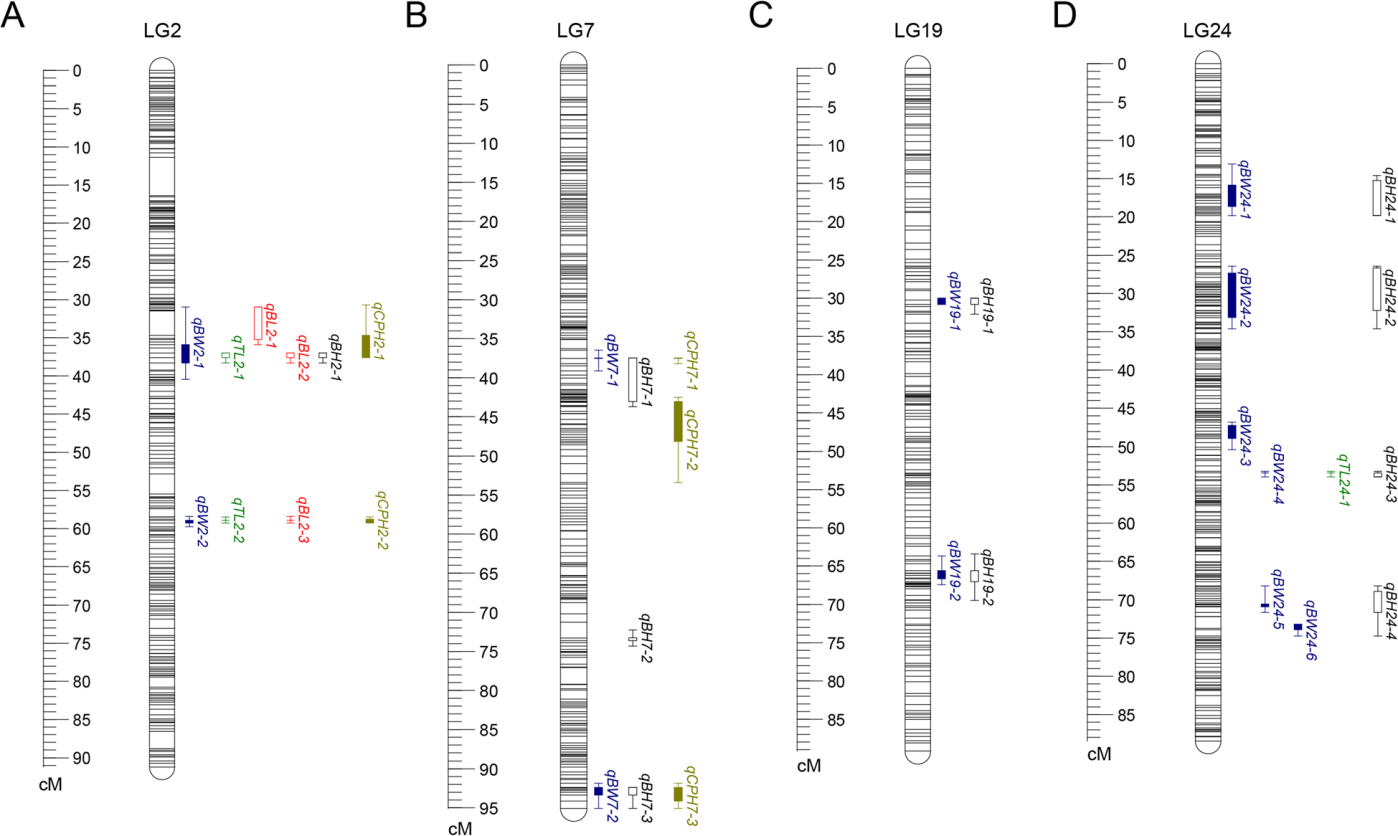
The distribution patterns of growth-related QTL clusters on LG2 (**a**), LG7 (**b**), LG19 (**c**), and LG24 (**d**)

QTLs associated with quantitative traits are generally not randomly distributed across chromosomal regions and chromosomes. Previous investigations had identified a set of QTLs in QTL clusters, which were defined by the presence of multiple QTLs associated with different or similar traits, respectively [39, 40]. In this study, a total of 10 QTL clusters were detected in LG2, LG7, LG19 and LG24 (Fig. 4 and Additional file 9: Table S5). We also noted that the QTLs located in certain clusters were associated with more than three growth traits, e.g., LG2-cluster-1 (30.708–40.473 cM) possessed six QTLs pertaining to all of the five growth traits; LG2-cluster-2 (58.402–59.763 cM) harbored four QTLs related to four growth traits (BW, TL, BL and CPH); LG7-cluster-1 (36.461–53.388 cM) possessed four QTLs significantly associated with three growth traits (BW, BH and CPH). Moreover, the analysis results indicated that QTL confidence intervals in LG2-cluster-2 or LG7-cluster-2, or LG24-cluster-3 displayed a high degree of overlapping with each other. Therefore we can make use of the overlapping regions to further analyze the gene annotation for obtaining more useful information.

### Candidate genes for growth

A total of 24 SNP markers located in the confidence intervals of body weight QTLs were selected and utilized to identify candidate growth-related genes. By means of searching against the *S. argus* genome using ddRAD-Tag sequences, the 50 kb regions flanking to each SNP marker were obtained from corresponding scaffolds. Based on the annotation information of genome, a total of 32 genes were located in these regions (Table 6). Among them, 15 potential growth-related genes were obtained, such as HMG box-containing protein 1 (*Hbp1*), centrosomal protein of 131 kDa (*Cep131*), nucleosome assembly protein 1-like 1 (*Nap1l1*), Ataxin-1 (*Atxn1*), serine/threonine-protein kinase pim-3 (*Pim3*), scm-like with four MBT domains protein 1 (*Sfmbt1*), prickle-like protein 2 (*Prickle2*), voltage-dependent calcium channel subunit alpha-2/delta-3 (*Cacna2d3*), transcription cofactor vestigial-like protein 4 (*Vgll4*), apoptotic protease-activating factor 1 (*Apaf1*), and PDZ domain-containing protein 4 (*Pdzd2*), which have been reported to play important parts in cell proliferation and growth, tumorigenesis, tumor suppressor, and muscle regeneration, respectively [41–54]. These candidate genes are worthy of further studies to reveal their exact roles in genetic control of growth traits in the future.

**Table 6:** Prediction of candidate genes based on SNP markers associated with growth traits

Marker	LG	Position (cM)	Scaffold	Annotation	Gene
R1_192243	LG2	58.917	scaffold1197	Protein shisa-6	*Shisa6*
R1_98424	LG2	37.607	scaffold339	Cell death-inducing p53-target protein 1	*Cdip1*
				MKL/myocardin-like protein 2	*Mrtfb*
R1_235502	LG5	42.973	scaffold1374	Apoptotic protease-activating factor 1	*Apaf1*
R1_269416	LG5	25.992	scaffold1410	Protein bicaudal D homolog 1-like	*Bicd1*
R1_99132	LG5	44.787	scaffold195	MANSC domain-containing protein 1	*Mansc1*
R1_275284	LG5	43.022	scaffold273	Probable tubulin polyglutamylase TTLL1	*Ttll1*
R1_151287	LG5	40.377	scaffold75	Serine/threonine-protein kinase pim-3	*Pim3*
R1_254692	LG5	40.042	scaffold86	Nucleosome assembly protein 1-like 1 isoform X3	*Nap1l1*
R1_205729	LG11	43.640	scaffold381	Fibronectin type-III domain-containing protein	*Fndc*
				LRRN4 C-terminal-like protein	*Lrrn4cl*
R1_113228	LG13	0.543	scaffold1603	Centrosomal protein of 131 kDa isoform X1	*Cep131*
				E3 ubiquitin-protein ligase RNF213	*RNF213*
				Nuclear protein localization protein 4 homolog	*Nploc4*
R1_232824	LG24	31.648	scaffold1004	Cadherin-22-like isoform X3	*Cad22l*
R1_119872	LG24	31.488	scaffold1050	Transcription cofactor vestigial-like protein 4	*Vgll4*
				Ubiquitin-like modifier-activating enzyme ATG7	*Atg7*
R1_216145	LG24	70.541	scaffold123	Tribbles homolog 1	*Trib1*
R1_269254	LG24	17.017	scaffold1587	Protein FAM19A1 isoform X1	*Fam19a1*
R1_79166	LG24	70.574	scaffold1590	LReO_3 protein	*LReO_3*
R1_287640	LG24	70.906	scaffold1611	ETS-related transcription factor Elf-3-like isoform X1	*Elf3l*
R1_50011	LG24	18.813	scaffold1648	Ataxin-1	*Atxn1*
				HMG box-containing protein 1-like	*HBP1*
R1_255417	LG24	18.653	scaffold225	Dynein heavy chain 1, axonemal	*Dnah17*
R1_115418	LG24	31.488	scaffold551	Guanine nucleotide-binding protein G(s) subunit alpha	*Galphas*
				Leucine zipper protein 2	*Lzp2*
				Tubulin beta chain-like	*Tubbl*
R1_137885	LG24	47.525	scaffold594	Voltage-dependent calcium channel subunit alpha-2/delta-3	*Cacna2d3*
R1_1854	LG24	18.813	scaffold619	Prickle-like protein 2	*Prickle2*
R1_219435	LG24	31.194	scaffold820	Phosphatidate cytidylyltransferase, mitochondrial	*Tam41*
R1_227285	LG24	73.712	scaffold839	PDZ domain-containing protein 4	*Pdzd2*
R1_129529	LG24	17.156	scaffold971	Protein kinase C, delta	*PKCΔ*
				Scm-like with four MBT domains protein 1	*Sfmbt1*

*LG* Linkage group

## Discussion

### ddRADseq and SNPs genotyping

As a keystone technology with ability to efficiently and accurately determine genotypes, RADseq and its derivative methods have brought in a revolution for the large-scale development of polymorphic SNPs, an excellent tool for population genomics and genetics studies [3, 11, 55]. With the increase of sequencing depth and cost reduction of NGS, RADseq has now been widely applied in construction of linkage maps and QTL fine mapping of genetic traits for commercial fishes. For example, genetic map and growth-related QTLs in turbot (6647 SNPs) [56], blunt snout bream (14,648 SNPs) [57], Yangtze River common carp (7820 SNPs and 295 SSRs) [3], golden pompano (12,358 SNPs) [20],Yellow River common carp (6230 SNPs and 65 SSRs) [1], and *P. ussuriensis* (7435 SNPs) [9]; genetic map and sex determination QTLs in hāpuku (1575 SNPs) [58], mandarin fish (3283 SNPs) [59], channel catfish (4768 SNPs) [60]; linkage map and disease resistance-related QTLs in Asian seabass (6425 SNPs) [61], Japanese flounder (12,712 SNPs) [16]; genetic map and fatty acid compositions-related QTLs in Asian Seabass (2424 SNPs) [62]. Accumulating achievement has reinforced the consensus among geneticist about the advantage of RADseq technology. In the present study for *S. argus*, ddRADseq-based SNPs discovery in a F1 full-sib family was performed for the first time. A total of 1753.7 M pair-end reads and 293,309 original RAD markers were generated, including 88,789 polymorphic SNP loci with a polymorphism ratio of 30.27%, which was higher than that found in common carp (21.1%) [12] and pikeperch (14.63%) [63]. According to our preliminary population genetic analysis (unpublished data), we have inferred that the higher polymorphism rate is due to a high genetic diversity of *S. argus*. Both the sufficient sequencing depths for each progeny and the genotype integrity of these SNP markers (> 90%) in the mapping population guaranteed a high-level of genotyping accuracy (Table 2) and finally, abundant high-quality Mendelian SNPs were retained as effective marker resource for linkage mapping. Our results once again demonstrated that ddRADseq is an effective approach for SNPs identification in linkage mapping researches.

### High-quality genetic map

A genetic linkage map with good quality is a basic genetic tool for fine QTL mapping, map-based gene cloning, and marker-assisted breeding [64]. This study reports the first genetic linkage map of *S. argus* covering 99.2% of genome, 6193 SNPs were assigned to 24 LGs (Fig. 1). A diploid chromosome number of 2 *N* = 48 was identified in *S. argus* by karyotype analysis [36], this result is in agreement with the number of LGs in this map, indicating that each LG corresponds to one chromosome and all *S. argus* chromosomes are covered by this genetic map. Nevertheless, the detailed correspondences require to be clarified in future exploration. Moreover, the female and male linkage maps were constructed. The results of synteny analysis showed that both female and male maps showed a high degree of syntenic relationship with the consensus map (Additional file 8: Figure S4), reflecting a high quality and reliability of the *S. argus* linkage maps. The average marker number of 258 per group and inter-marker distance of 0.35 cM suggested that the consensus map can provide a reference to help position sequence scaffolds on the physical map, which would be beneficial for identifying genes near each SNP locus.

A number of HD genetic maps have already been developed in aquaculture fishes. Compared with most of these linkage maps, the final *S. argus* genetic map containing thousands of SNPs has an improved number of markers per linkage group, a shorter average marker interval, and longer ddRAD marker sequences. Meanwhile, there were improvements in density and resolution of *S. argus* map in comparison to maps constructed using RADseq methods in some other fishes, e.g., blunt snout bream *Megalobrama amblycephala* (237 SNPs per LG, 0.56 cM) [57], mandarin fish *Siniperca chuatsi* (137 SNPs per LG, 0.61 cM) [59], crucian carp (170 SNPs per LG, 0.44 cM) [65] and bighead carp *Hypophthalmichthys nobilis* (130 SNPs per LG, 0.75 cM) [66]. In Japanese flounder genetic map, although much more markers (12,712 SNPs) were assigned to 24 consensus LGs, a lower average marker interval (0.47 cM) was found [16]. However, when compared to several ultra-high-density linkage maps consisting of tens of thousands markers, such as maps of *S. salar* [67] and *Ictalurus punctatus* [60, 68, 69], further improvements for the *S. argus* map are absolutely necessary. Nevertheless, as the first HD linkage map for *S. argus* and even for family Perciformes, this map not only can enable the fine-mapping of QTLs for important traits, but it also can provide useful tools for genetic and genomic studies in both *S. argus* and closely related species.

RAD markers are generally not randomly distributed across LGs, both marker-dense regions and some marker deserts can be clearly observed in linkage maps [70]. In our study, gaps shorter than 5.0 cM accounted for an average of 98.0% of gaps. Although the average distance between adjacent markers was short (0.35 cM), gaps greater than 5.0 cM were not restricted to a particular group and were found on seven LGs (Table 4). This pattern may be one of the chief reasons for the non-random distribution of markers, as well as uneven marker polymorphism and recombination rates between female and male parents [12]. In the coming research, changing the types of restriction enzyme for RAD library construction or increasing the size of mapping population may be the alternative approaches to improve the *S. argus* genetic map.

### Comparative genome analysis

Comparative mapping is an approbatory route for gene localization and function prediction, QTL identification, and genome evolution exploration in non-model fishes [9]. Here we analyzed the syntenic relationships between the *S. argus* genetic map and the genome of 10 teleosts. A relatively high-level of genomic synteny between *L. crocea* and *S. argus* were observed, about 15.0% of map markers had homologs in the *L. crocea* chromosomes (Fig. 2b). Most *S. argus* LGs and *L. crocea* chromosomes exhibited a high degree of syntenic relationship, there was only one *S. argus* LG (LG19) with a one-to-two relationship with *L. crocea* chromosomes (chr8 and chr16). Moreover, 13.0% of the homologous markers were located on various *L. crocea* chromosomes instead of conserved syntenic blocks, indicating a measure of intra-chromosomal rearrangements between *S. argus* and *L. crocea*. A similar phenomenon has been reported in the comparative analysis between southern catfish and channel catfish [71]. As a common chromosomal rearrangement mode in teleosts, translocation exists in many fishes. Translocation rates were believed to be positively correlated with lineage relationships between two fishes [72]. We also observed that each of two *O. niloticus* chromosomes was homologous with two *S. argus* LGs (chr7 with LG1 and LG16, chr21 with LG4 and LG9) (Fig. 2c). According to the hypothesis of previous studies, the ancestor of teleosts had the original chromosome number of 24, chromosomal fission and fusion played important parts in the formation of various chromosome numbers in fishes [73, 74]. Therefore, it could be inferred that two ancestral chromosomes of *O. niloticus*, which were homologous with LG1 and LG16, LG4 and LG9 of *S. argus*, respectively, fused and formed 22 chromosomes gradually during evolution.

### QTL mapping and candidate genes for growth traits

A few preliminary studies on growth regulation have been reported in *S. argus* [75–77], but the principal components of growth traits QTL were still not investigated. Economically valuable traits of domestic animals are believed to be quantitative traits controlled by multiple loci or genes located in different genome regions [78]. In the present study, the HD linkage map enabled us to determine the number and position of the QTLs for growth traits. A number of significant QTLs associated with five growth traits were identified on different LGs (Table 5), indicating that growth-related traits are controlled by multiple QTL loci or regions in *S. argus*. Similar results were found in some other fish species and multiple QTLs were identified for one trait, e.g., 10 QTLs for body weight were detected on two LGs of *P. ussuriensis* [9], 14 QTLs for body height of *T. blochii* were were detected on two LGs [20], 4 QTLs for head length of common carp were detected on four LGs [3], and 5 significant QTLs for body length were detected on three LGs in *Sander lucioperca* [63]. These findings suggest that the growth traits are regulated by numerous loci or genes in fish, a few of these loci may have major effects and the remaining loci may possess minor effects. Moreover, the QTL regions were generally concentrated on certain groups (LG2, LG7 and LG24), suggesting that genes from different chromosomes may contribute to the same trait.

The phenotypes of five growth traits were found to be highly correlated with each other (Table 1). Interestingly, QTLs for these traits showed similar distribution patterns as expected and were mainly clustered into several main regions on *S. argus* genetic map. Many QTLs located in six clusters were associated with at least three growth traits, and several highly-overlapped QTL regions for BW and BH were detected on four LGs (Fig. 4), reflecting that these genomic regions may have key roles in growth regulation. Multifactorial QTLs for growth traits have also been detected in common carp [1, 3], *L. calcarifer* [18], *S. chuatsi* [59], *S. lucioperca* [63], bighead carp [66] and so on, implicating the pleiotropic effect of a QTL for multiple traits. Based on critical factors that control various traits through diverse metabolic pathways, pleiotropy may contribute to multifactorial QTLs [4]. It is generally believed that when closely-linked markers in multifactorial QTL regions were utilized in MAS, several correlated traits could be improved simultaneously. Thus, these QTL clusters will effectively facilitate the selective breeding of *S. argus*. However, our study is just the first attempt to locate growth-related QTLs, further verifications are needed to improve the scale and quality of identified QTLs.

A total of 32 candidate genes were identified in reference genome around the SNP markers located in the confidence intervals of QTLs related to body weight (Table 6). Among them, 15 genes are most likely to have some associations with growth, which indicated by the previous function studies in human or other mammalian species. Growth in most tissues is generally controlled by two different patterns: cell division and cell size increasing. *Hbp1* is a transcription factor and a potent cell cycle inhibitor in normal and cancer cells, it activates or represses the expression of different cell cycle genes through direct DNA binding, cofactor recruitment, chromatin remodeling, or neutralization of other transcription factors [41]. *Sfmbt1* acts as a key regulator affecting keratinocytes proliferation and apoptosis [42], and common deletions in this gene shows a significant association with fasting plasma glucose, implicating certain functions in some metabolic-related traits [43]. *Vgll4* regulates muscle regeneration as a negative regulator of Yes-associated protein (YAP) in mice [44]. It is also a transcriptional cofactor acting as a novel tumor suppressor to inhibit cancer progression [45–47]. Current researches have revealed that *Pim3*, *Cep131*, *Atxn1*, and *Nap1l1* play potential roles in tumorigenesis. *Pim3* is correlated with enhanced tumor growth and cell survival [48] and its overexpression can enhance the proliferation of hepatoblastoma cells [49]. *Cep131*, a regulator for centrosome duplication and genome stability, can function as an oncogene and promote cell proliferation and migration in hepatocellular carcinoma [50]. *Atxn1* has been demonstrated to be involved in tumorigenesis of cervical cancer cells via the EGFR-RAS-MAPK signaling pathway [51]. Whereas, *Nap1l1* can promote the proliferation of induced pluripotent stem cells [52]. In addition, *Prickle2* and *Cacna2d3* are two potential tumor suppressor genes in the development and progression of carcinoma [53, 54]. However, it should be aware of the fact that these genes are not necessarily the causative genes. Further studies are still needed to clarify the real associations between gene polymorphisms and growth traits in *S.argus*.

## Conclusions

A high-density genetic linkage map with 6193 SNPs derived from ddRADseq data was constructed for *S. argus* with an average marker interval of 0.35 cM. Comparative mapping generates deeper insights into genomic evolution of *S. argus* by revealing a high level of syntenic relationship between this map and *L. crocea* genomes. A total of 44 significant QTLs for growth-related traits were identified on 11 LGs, and 19 of which were associated with body weight. Fifteen genes playing potential parts in cell proliferation and growth, tumorigenesis and muscle regeneration were recognized from some genome regions in QTL intervals. The *S. argus* genetic map will act as a useful tool in future genetic, genomic and evolutionary studies. These QTLs, SNP markers and candidate genes associated with growth traits lay the foundation for further genetic dissection of growth and can accelerate the marker-assisted breeding researches for genetic improvement in important traits of *S. argus*.

## Methods

### Mapping family and phenotypic data

In late May 2016, two wild *S. argus* populations were collected from Zhuhai offshore area (21.50°N, 113.15°E; *N* = 46) and Taiwan Strait (23.32°N, 120.60°E; *N* = 31). In August, a F1 full-sib family comprised of ~ 8 thousand larvae was produced by crossing of a dam from Taiwan Strait population and a sire from Zhuhai population. The larvae had been raised in a 5 m × 5 m × 2 m tank for 40 days. The juvenile offspring were then transferred and raised in a 0.3 ha disinfected muddy pond (water salinity was 15 ± 5 ‰) at YuCheng Fish Fry Culture Farm in Zhuhai, Guangdong, China. The experimental fish were fed twice at 8:00 am and 6:00 pm each day. The fishery feed was purchased from YueHai Feeds Group Company in Guangdong, China. After 13 months of culture, a total of 420 progeny were randomly sampled for phenotypic measurements. Alive fish were anesthetized by immersing them in a tricaine methanesulfonate (MS-222) bath (300 mg/L), then phenotypic parameters of eight growth-related traits including body weight (BW), total length (TL), body length (BL), body height (BH), pre-dorsal length (PD), pre-anal length (PA), head length (HL) and caudal peduncle height (CPH) were measured and recorded. The distribution patterns (normal or non-normal) of phenotypic values of growth traits were determined by the Kolmogorov-Smirnov tests. To investigate the relationships among growth-related traits, Pearson’s correlation coefficients (*r*) were calculated. The statistical analyses were performed with the SPSS 19.0 software package (IBM, Armonk, NY, USA). Of the 420 progeny sampled, 200 individuals were randomly selected and subjected to genetic maps construction and growth-related QTL mapping. Fin clips of the two parents and full-sib progeny were excised and preserved in absolute ethanol at − 20 °C until DNA isolation. Genomic DNA was extracted from fin clip tissues using a published traditional phenol-chloroform method with minor modification [79], its quality was evaluated using a Qubit fluorometer (Invitrogen, USA) and 1.0% agarose gel electrophoresis. DNA samples with high purity (A_260_/A_280_ = 1.8 ~ 2.0, A_260_/A _230_ = 1.8 ~ 2.2) and good integrity (the primary band size > 20 kb) will be utilized for the construction of ddRAD libraries. All DNA samples were adjusted to a concentration of 50 ng/μL by diluting with 10 mM Tris-EDTA buffer. After sampling, the fish used in this study were still cultured in the muddy pond for further investigation.

### ddRAD library construction and sequencing

In this study, a total of 202 genomic DNA samples from the two parents and their 200 offspring were used to construct ddRAD libraries by following the previously described protocol [11]. Briefly, 500 ng of genomic DNA from each individual was double-digested with 20 U of restriction enzymes *Eco*RI and *Nla*III (New England Biolabs, Ipswich, MA, USA) in a 50 μL reaction system at 37 °C for 30 min. The digested product was heat-inactivated for 20 min at 65 °C, and then, was purified using a Qiagen MinElute Reaction Cleanup Kit (Qiagen, Valencia, CA, USA). The digested fragments were ligated with P1 [a unique 4–8 bp MID (multiplex identifier) for distinguishing each individual] and P2 adapters at 16 °C over night. Whereafter, the ligated fragments were amplified (15 cycles of amplification) with High-Fidelity DNA Polymerase (Thermo Scientific, USA) using a set of primers that introduce sample specific barcode and sequencing primers. The PCR products were purified and size-selected (400–600 bp) via retrieval from an agarose gel. After insert-size analysis, the libraries were pooled with equal amount to prepare the final library that was sequenced in a lane of the Illumina HiSeq2500 platform (Illumina Inc., San Diego, CA, USA). In order to enhance sequencing coverage and improve genotyping accuracy, samples were merged in pools of 15 ~ 16 samples for sequencing and genotyping the offspring, while the two parents were genotyped in a single pool, respectively. Overall, 15 pools (2 for the parents and 13 for the offspring) were sequenced with 150 bp pair-end reads.

### Quality control and SNP genotyping

Firstly, sequence reads for each individual were extracted and separated according to the specific MIDs using an in-house Perl script. The short reads without sample-specific MIDs and expected restriction enzyme motifs were discarded. Based on the quality score, the raw sequencing data were filtered with the Illumina read trimming tool Trimmomatic v0.39 [80] by removing adapters, the reads with bases quality below a threshold of 3, and the reads with average Phred quality per base below 15. The resulting high-quality reads were then subjected to subsequent analyses. To identify loci in a set of individuals, the STACKS software (version 2.0 Beta 7; http://creskolab.uoregon.edu/stacks) [81] was utilized to de novo assemble the loci from the sequencing data for SNP calling. The libraries of loci were created by USTACKS, CSTACKS, SSTACKS and GENOTYPE, i.e., one for each individual and one for all the loci shared among the individuals. Minimum depth of coverage required to create a stack is 3; Maximum distance allowed between stacks is 3; 2 mismatches allowed between sample loci when build the catalog. Previously described parameters [59] were adopted in this study.

### Linkage map construction

SNP loci that were heterozygous in at least one parent and genotyped in at least 90% of all the progeny were considered as high-quality markers and retained for further map construction. Based on their segregation patterns, SNP markers were categorized into three types: 1:1 (type lm × ll or nn × np), 1:2:1 (type hk × hk) and 1:1:1:1 (type ab × cd or ef × eg). Linkage maps were constructed using JoinMap 4.1 [82] under the CP (cross pollinators) algorithm in this study. Chi-square tests were performed to examine the Mendelian segregation pattern of each SNP locus using the “Locus genot. Freq.” function at the confidence level of 0.01. Markers with significant segregation distortion were excluded from linkage mapping analysis (*P* < 0.01). The linkage relationship between markers was examined by estimating logarithm of the odds (LOD) scores for recombination fraction. Linkage group assignments were made under a LOD threshold of 8.0. The regression mapping algorithm was selected for linkage mapping with the following settings: Rec = 0.45, LOD = 1.0, Jump = 5. Recombination frequencies were converted into map distances in centiMorgans (cM) using the Kosambi’s mapping function. The estimated genome length (G_e_) was the average of lengths calculated by two previously described methods [37, 38]. The resulting linkage maps were graphically visualized using MapChart 2.2 software [83].

### Comparative genomics

ddRAD marker sequences (142–146 bp in length) were used as a query for homology searches against available fish genomes using BLASTn v.2.8.1+ (ftp://ftp.ncbi.nlm.nih.gov/blast/executables/blast+/LATEST) with an e-value cutoff of 1e-10. Reference sequences of *Danio rerio* (GRCz11), *Oryzias latipes* (ASM223469v1), *Tetraodon nigroviridis* (ASM18073v1), *Gasterosteus aculeatus* (ASM18067v1), *O. niloticus* (UMD1), *Fugu rubripes* (FUGU5), *Ietalurus punetaus* (IpCoco_1.2), *Larimichthys crocea* (ASM384579v1), *P. olivaceus* (ParOli_1.1), and *S. salar* (ICSASG_v2) were downloaded from the NCBI Genome Assembly database (ftp://ftp.ncbi.nlm.nih.gov/genomes). Only ddRAD loci present in the linkage map and their putative homologs in the other fish genomes were taken into account in further analyses. For sequences that had multiple hits, only the ones with the lowest e-value were treated as their homologs and retained for comparative analysis. The final genomic synteny was visualized using the Circos software [84] release 0.69 (http://circos.ca/software/download/).

### QTL mapping for growth traits

In this study the MapQTL 5.0 software [85] was employed to identify QTLs associated with growth traits following the mapping method of multiple QTL model (MQM). Regression algorithm was used for mapping quantitative trait loci in line crosses. Significant associations between loci and growth traits were detected with a mapping step size of 1.0 cM, and cofactors for MQM were automatically selected with a *p*-value of 0.05. Both genome-wide and chromosome-wide significant LOD thresholds were estimated using 1000 permutation tests with a confident interval of 95%. The location of each QTL was determined according to its LOD peak location and surrounding region. The percentage of the phenotypic variance explained by a QTL was estimated at the highest probability peak. Finally, the QTL profiles were visualized with MapChart 2.2 [83]. To identify candidate growth-related genes, ddRAD-tag sequences corresponding to SNP markers that were significantly correlated with growth traits were mapped on the *S. argus* reference genome (unpublished data) by BLASTn. Coding gene sequences were extracted from a 50 kb region containing 25 kb upstream and 25 kb downstream sequences flanking to each SNP locus. The gene functions were predicted subsequently based on the annotation information of genome. In addition, these gene sequences were also searched against the NCBI non-redundant (Nr) protein database to verify their annotations using BLASTx with an e-value cut-off of 1.0 × 10^− 10^.

## Supplementary information

**Additional file 1 **: **Table S1.** The statistics of eight growth-related traits of *S. argus* full-sib family. BW: body weight; TL: total length; BL: body length; BH: body height; PD: pre-dorsal length; PA: pre-anal length; HL: head length; CPH: caudal peduncle height; SD: standard deviation; CV: coefficient of variation.

**Additional file 2 **: **Fig. S1.** Frequency distribution of phenotypic values of eight growth-related traits of *S. argus* full-sib family. BW: body weight (g); TL: total length (cm); BL: body length (cm); BH: body height (cm); PD: pre-dorsal length (cm); PA: pre-anal length (cm); HL: head length (cm); CPH: caudal peduncle height (cm).

**Additional file 3 **: **Table S2.** Segregation patterns of heterozygotic SNP locus in the mapping population of *S. argus*.

**Additional file 4 **: **Table S3.** Statistic information of the sex-specific genetic maps of *S. argus.*

**Additional file 5 **: **Fig. S2.** Illustration of female linkage map for *S. argus*. The map demonstrates the genetic lengths and marker distribution of 3512 SNP loci along the 24 linkage groups (LG1f-LG24f). The linkage groups are displayed by the vertical bars with black lines in each linkage group indicating a marker position. Genetic distance is shown by the vertical scale line with centiMorgans (cM).

**Additional file 6 **: **Fig. S3.** Illustration of male linkage map for *S. argus*. The map demonstrates the genetic lengths and marker distribution of 3627 SNP loci along the 24 linkage groups (LG1m-LG24m). The linkage groups are displayed by the vertical bars with black lines in each linkage group indicating a marker position. Genetic distance is shown by the vertical scale line with centiMorgans (cM).

**Additional file 7 **: **Table S4.** Estimation of the expected genetic distance of *S. argus* linkage maps.

**Additional file 8 **: **Fig. S4.** Circos diagram representing the syntenic relationships between the consensus map and sex-specific maps of *S. argus*. (a) syntenic relationships between consensus map (right) and female map (left); (b) syntenic relationships between consensus map (right) and male map (left). Each colored arc represents a marker match between the linkage groups of two maps.

**Additional file 9 **: **Table S5.** QTL clusters for growth-related traits in *S. argus*.

## Data Availability

The datasets supporting the findings of this article are included within the article and its supplementary information files. The raw sequencing data were deposited at the NCBI Sequence Read Archive (SRA) with the accession Number PRJNA578058.

## Abbreviations

AFLP: Amplified fragment length polymorphism
GBS: Genotyping-by-sequencing
LGs: Linkage groups
LOD: Logarithm of odds
MAS: Marker-assisted selection
NGS: Next-generation sequencing
PVE: Phenotypic variance explained
QTL: Quantitative trait loci
RAD: Restriction site-associated DNA
RAPD: Random amplified polymorphic DNA
SNP: Single nucleotide polymorphism
SSR: Simple sequence repeat
