# Supplementary figures and images for: ddRADseq-assisted construction of a high-density SNP genetic map and QTL fine mapping for growth-related traits in the spotted scat (Scatophagus argus)
Source: BMC Genomics. 2020 Apr 3;21:278. doi: 10.1186/s12864-020-6658-1 (PMC7126399; doi:10.1186/s12864-020-6658-1)

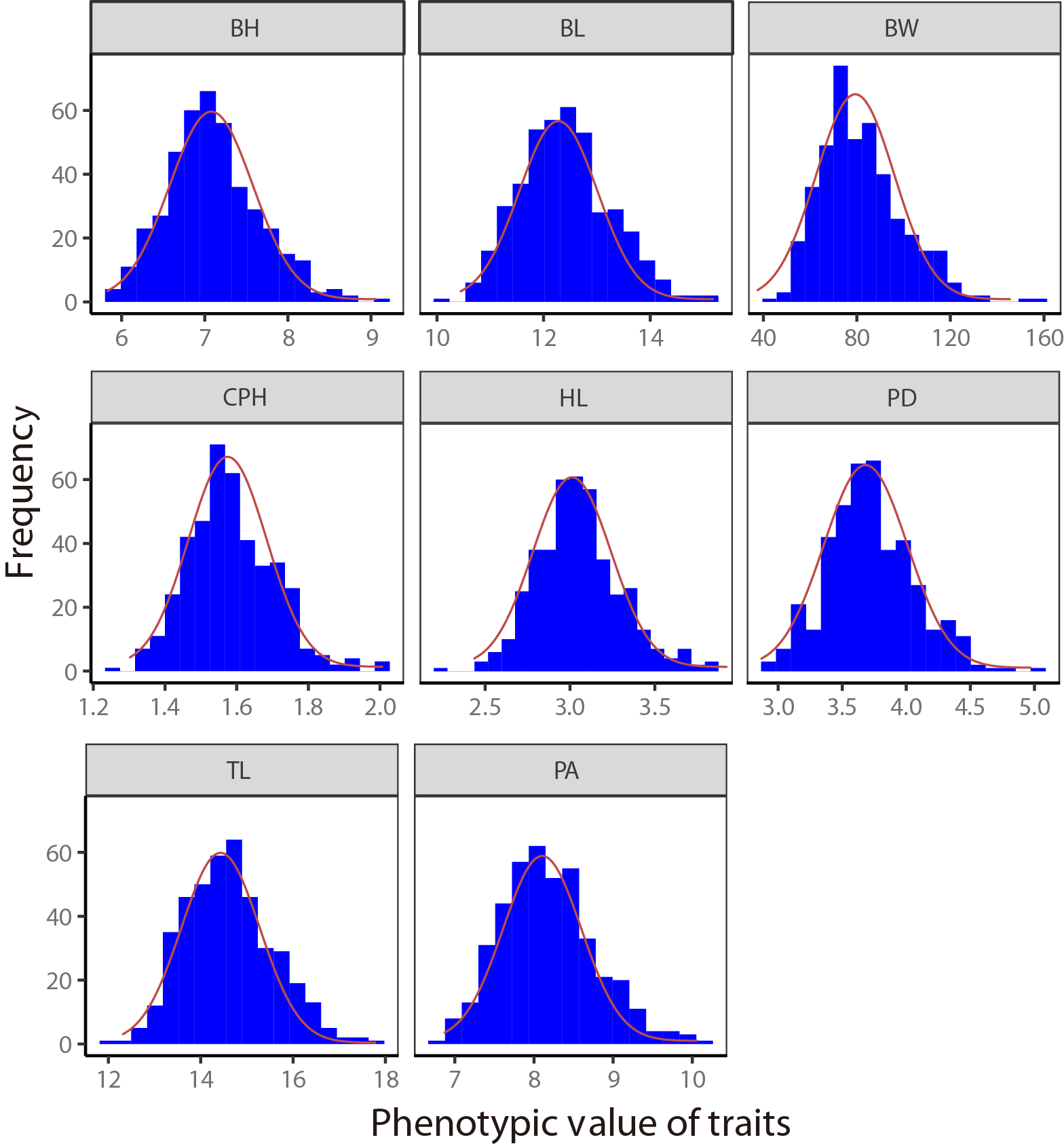

Supplement: Supplementary file 2 — Additional file 2 : Fig. S1. Frequency distribution of phenotypic values of eight growth-related traits of S. argus full-sib family. BW: body weight (g); TL: total length (cm); BL: body length (cm); BH: body height (cm); PD: pre-dorsal length (cm); PA: pre-anal length (cm); HL: head length (cm); CPH: caudal peduncle height (cm). [file 12864_2020_6658_MOESM2_ESM.tif]

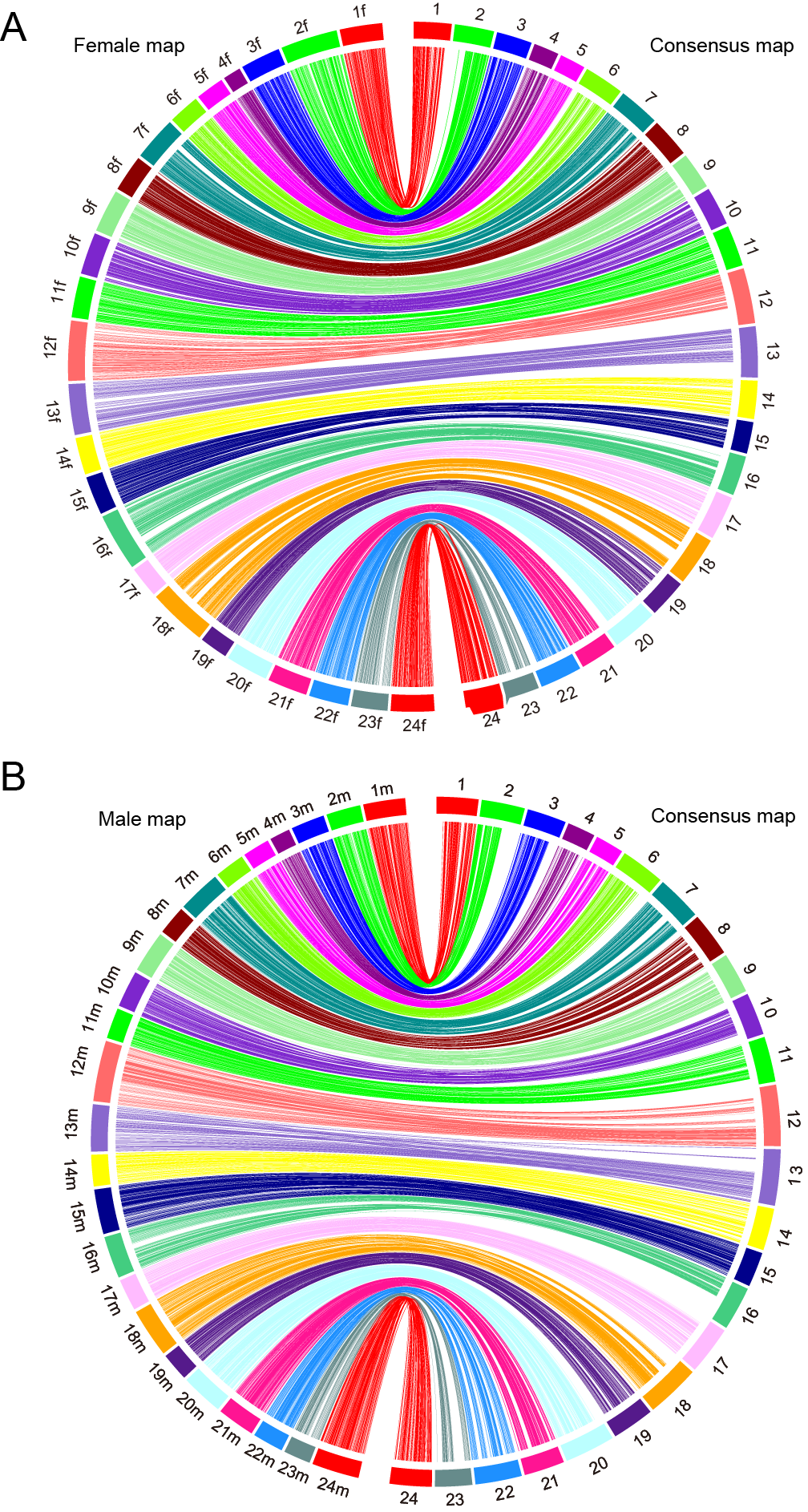

Supplement: Supplementary file 8 — Additional file 8 : Fig. S4. Circos diagram representing the syntenic relationships between the consensus map and sex-specific maps of S. argus. (a) syntenic relationships between consensus map (right) and female map (left); (b) syntenic relationships between consensus map (right) and male map (left). Each colored arc represents a marker match between the linkage groups of two maps. [file 12864_2020_6658_MOESM8_ESM.tif]
